# Right versus left transthoracic approach for lymph node-negative esophageal squamous cell carcinoma

**DOI:** 10.1186/s13019-015-0328-4

**Published:** 2015-09-18

**Authors:** Qilong Ma, Wengao Liu, Hao Long, Tiehua Rong, Lanjun Zhang, Yongbin Lin, Guowei Ma

**Affiliations:** Sun Yat-sen University Cancer Center, Guangdong Esophageal Cancer Institute, State Key Laboratory of Oncology in South China, 651 Dongfengdong Road, 510060 Guangzhou, China

**Keywords:** Transthoracic approach, Lymph node-negative, Esophageal cancer, Survival analysis

## Abstract

**Background:**

To compare the right and left transthoracic approach on the post-operative survival of patients with lymph node-negative esophageal squamous cell carcinoma.

**Methods:**

Six hundred and ninety-five ESCC patients who underwent esophagectomy between 1990 and 2005 were retrospectively enrolled in the present study and were confirmed by histology to be of no lymph node metastasis. Those who had received neoadjuvant chemotherapy or radiotherapy were excluded from the study. Patients were divided into two groups, the left (*n* = 545) and right (*n* = 150) transthoracic groups. The follow-up duration ranged from 1 to 20 years with a mean of 7 years. Kaplan–Meier and univariate and multivariate Cox proportional hazards were used for analysis.

**Results:**

3- and 5-year CSS rates were 62.0 % and 44.0 % in the left group, while the corresponding figures in the right group were 56.0 % and 40.0 %(*P* < 0.05). The overall survival for the two groups was significantly different (*P* = 0.045). Survival analyses were stratified by stages, which found that the favorable survival advantage was not present. When the survival curves were stratified by tumor locations, a significant difference was not revealed. Surgical approaches were regarded as one of the prognostic factors in the univariate analysis (*P* = 0.019). However, this significance could not be confirmed in multivariate Cox regression analysis (*P* = 0.193).

**Conclusions:**

The left transthoracic approach is superior in some aspects to the right transthoracic approach regarding surgical and oncological outcomes in the treatment of lymph node negative ESCC.

## Background

Esophageal cancer (EC) is the eighth most common cancer and the sixth leading causes of cancer death worldwide [1]. The incidence of esophageal cancer, especially adenocarcinoma, has been on the increase in western countries [2]. In China, squamous cell carcinoma is the commonest histological subtype and accounts for over 95 % of the cases [3, 4].

Surgery is the single most effective treatment with curative intent for esophageal carcinoma [5]. The commonest open surgical approaches include transthoracic and transhiatal esophageal resection. The transthoracic esophageal resection includes Ivor Lewis (laparotomy and right thoracotomy), McKeown (right thoracotomy, laparotomy, and neck incision) and left transthoracic esophagectomy [6, 7]. We categorized these surgical approaches as left and right transthoracic approaches in our study.

A review of the Medicare database of the United States [8] showed that the mortality rates following esophagectomy ranged from 3.2 % to 6.1 %, and the complication rates varied from 30 % and 80%with an average of 50 % [9]. However, the right transthoracic esophagectomy is preferred to that of the left especially in the western society because it provides excellent surgical exposure to the esophagus and regional lymph nodes. Despite this, the left transthoracic technique still serves as an effective alternative and is being advocated by many surgeons. Whether or not there is a difference regarding oncological outcome between the two approaches is not conclusive. Therefore, we well designed this study to compare thoroughly the clinical outcomes between the two groups for lymph node-negative esophageal squamous cell carcinoma (ESCC).

## **Methods**

This retrospective study was performed by utilizing a database established at the Sun Yat-sen University Cancer Center, Guangzhou, China. We enrolled 695 ESCC patients who underwent esophagectomy in the Department of Thoracic Surgery between 1990 and 2005 and were confirmed by histology to be of no lymph node metastasis. Those who had received neoadjuvant chemotherapy or radiotherapy were excluded from the study. Patients were divided into two groups, namely, the left (*n* = 545) and right (*n* = 150) transthoracic groups. The follow-up duration ranged from 1 to 20 years, with a mean of 7 years. This study was approved by the Ethics Committee of Sun Yat-sen University Cancer Center.

The demographic data, surgical and oncological outcome were obtained from the established database. Baseline factors included sex, age, smoking history, alcohol consumption history, preoperative hemoglobin level, surgical duration, anastomosis method, tumor size, location, stage, and grade. Cancer staging was based on the American Joint Committee on Cancer (AJCC) staging manual (7th Edition) [10]. Because all the surgeries were completed before the publication of AJCC staging manual (7th Edition), it was challenging to verify the exact tumor locations according to the new criteria suggested by this version of staging manual. Fortunately, the distances from the superior incisor to esophageal lesions were well measured and recorded since every patient received gastroscopy routinely, and there figures could be used to estimate the tumor locations. In our study, tumors 15–20 cm distal to the superior incisor were considered as cervical location, while those 20–25 cm, 25–30 cm, and 30–40 cm distal to the incisor were considered as upper, middle , and lower thoracic locations, respectively. Survival time was defined as the time from surgery to death. To ensure that deaths were exclusively cancer-related, the patients who either died from other causes or were still alive at last follow-up were censored.

All the data were analyzed using SPSS Statistics Software (version 16.0, IBM SPSS, Inc.). The two-tailed Kruskal-Wallis H test was used to obtain *P* values. The 3-year and 5-year cancer-specific survival (CSS) rates were obtained and compared by Life Table Analysis. Survival curves were generated, and the log rank test was used to determine the statistical significance of the difference between the two groups. Stratification analysis was applied to investigate further the influence of surgical approaches on ESCC of different stages and locations. *P* value of less than 0.05 was considered to be statistically significant.

### The left thoracic approach

The patient was placed in the right lateral decubitus position. A traditional posterolateral incision was made along the sixth intercostal space in the left hemithorax. Mediastinal regional lymph nodes were resected in en bloc fashion with anatomical dissection of the esophagus. The gastric conduit was then harvested via transdiaphragmatic approach along with abdominal lymph node clearance. Gastro-esophageal anastomoses were constructed in the thorax of 518 (95.0 %) patients and on the neck of27 (5.0 %) patients.

### The right thoracic approach

Firstly, the patient was placed in left lateral decubitus position. A standard right posterolateral thoracotomy was placed along the fifth intercostal space in the right hemithorax. The esophagus along with the regional lymph nodes in the mediastinal region was removed. Then, the patient was re-positioned in a supine position. A second upper midline laparotomy was performed from the umbilicus to the xyphoid. With preservation of the right gastroeploic artery, the gastric conduit was harvested along with regional lymph nodes clearance in the abdominal region. After a third incision had been made extending along the sternocleidomastoid muscle 6 cm cephalad from the sternal notch through the platysma, the stomach was then drawn up to the neck through the chest. Finally, gastro-esophageal anastomoses were constructed in the left neck of 134 (89.3 %) patients. For the remaining 16 (10.7 %) patients who received Ivor-Lewis approach, their gastro-esophageal anastomoses were created in the right pleural cavity.

## Results

### Patient and surgical characteristics

Six hundred and ninety-five ESCC patients were enrolled in this study, of which 545 (78.4 %) and 150 (21.6 %) patients underwent the left and right transthoracic approaches, respectively. 70.8 % of the patients (n = 492) were male while 29.2 % (n = 203) were female. Mean age was 55.7 years in the left group and 56.9 years in the right group (*P* = 0.192). In the left group, most patients’ (74.3 %, n = 405) lesions located at the middle third of the esophagus, followed by tumors in the lower third of the esophagus (22.9 %, n = 125); whilst most patients’ lesions in the right group sat at the middle third of the esophagus (53.3 %, n = 80), followed by tumors in the upper third of the esophagus (42.0 %, n = 63, overall *P* < 0.001). Baseline characteristics of our cohort are summarized in Table 1. Operation durations were significantly different between the left and right transthoracic approaches with the mean time of 189 and 270 min respectively (*P* < 0.001). Greater intraoperative blood loss was observed in the right transthoracic group compared to the left group (*P* < 0.001). The incidence of postoperative complications was significantly higher in the former (26.7 % vs. 13.4 %, *P* < 0.001). In particular, higher chance of anastomotic leak (*P* < 0.001), incision infection (*P* < 0.001), and respiratory complications (*P* = 0.044) in the right transthoracic group were demonstrated in our study (Table 2). There were no significant differences regarding gender, age, smoking history, alcohol consumption history, preoperative hemoglobin level, tumor location, size, stage, and grade between the two groups.

**Table 1:** Baseline characteristics of patients grouped by surgical approach

Characteristic	Left transthoracic approach (n = 545)	Right transthoracic approach (n = 150)	P
Sex			0.995
Male	386 (70.6 %)	106 (70.7 %)	
Female	159 (29.4 %)	44 (29.3 %)	
Average age	55.66 ± 9.85	56.91 ± 8.42	0.192
Smoking history	324 (59.4 %)	89 (59.3 %)	0.980
Drinking history	109 (20.0 %)	28 (18.7 %)	0.716
Preoperative hemoglobin	134.33 ± 18.65	132.49 ± 17.38	0.222
Preoperative FEV1	2.42 ± 3.50	2.37 ± 0.70	0.086
Duration of surgery	189.24 ± 50.41	270.83 ± 68.38	<0.001
Blood loss during surgery	257.79 ± 126.56	297.96 ± 129.00	<0.001
Location of anastomosis			<0.001
Cervical	27(5.0 %)	134 (89.3 %)	
Intrathoracic	518 (95.0 %)	16 (10.7 %)	
Length of tumor	4.81 ± 1.96	4.67 ± 1.79	0.288
Pathological stage			0.336
Ia	48 (8.8 %)	11(7.3 %)	
Ib	258 (47.3 %)	67 (44.7 %)	
IIa	239 (43.9 %)	72 (48.0 %)	
Location of tumor			<0.001
Upper third	15 (2.8 %)	63 (42.0 %)	
Middle third	405 (74.3 %)	80 (53.3 %)	
Lower third	125 (22.9 %)	7 (4.7 %)	
Method of anastomosis			0.006
Manual	50 (9.2 %)	124 (82.7 %)	
Mechanical	495 (90.8 %)	26 (17.3 %)	
Grade of differentiation of tumor			0.102
High	213 (39.1 %)	44 (29.3 %)	
Middle	219 (40.1 %)	74 (49.3 %)	
Low	113 (20.7 %)	32 (21.3 %)	
Number of resected LNs^a^	9.18 ± 5.48	9.61 ± 8.02	0.171
Postoperative complications	73 (13.4 %)	40 (26.7 %)	<0.001

FEV1 forced expiratory volume in 1 second

LNs lymph nodes

**Table 2:** Incidence of postoperative complications in ESCC patients treated by the left or right transthoracic approach

Complication	Left approach group [number(%)]	Right approach group [number(%)]	*P*
Anastomotic fistula	10 (1.8)	18 (12.0)	<0.001
Anastomotic stenosis	14 (2.6)	7 (4.7)	0.155
Incision site infection	2 (0.4)	8 (5.3)	<0.001
Injury of recurrent laryngeal nerves	4 (0.7)	4 (2.7)	0.075
Chylothorax	3 (0.6)	2 (1.3)	0.303
Pneumothorax	12 (2.2)	3 (2.0)	0.574
Complication of respiratory tract	7 (1.3)	6 (4.0)	0.044
Complication of cardiovascular system	5 (0.9)	2 (1.3)	0.477

### Survival analysis

**Fig. 1:**
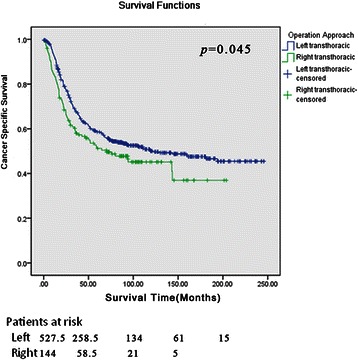
Overall survival curves. The cancer specific survival for the two groups was significant different (*P* = 0.045)

**Fig. 2:**
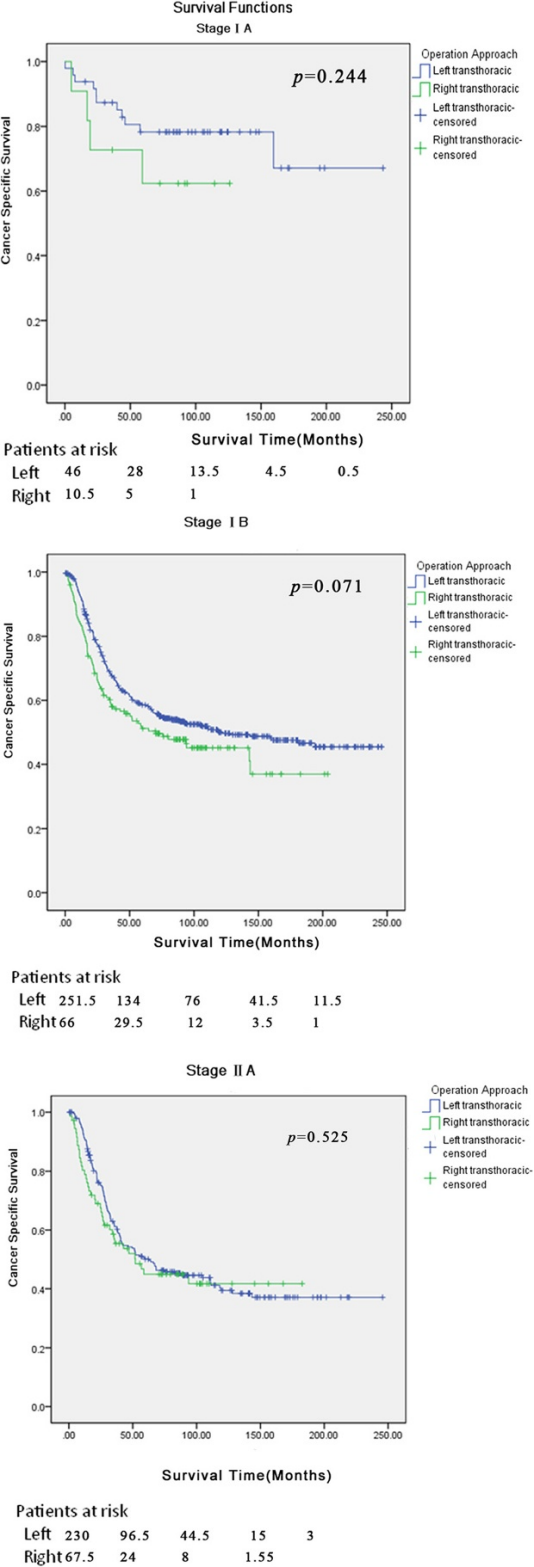
Cancer-specific survival curves stratified by tumor stage. Survival analyses were further stratified in terms of tumor stages, which found that the favorable survival advantage was not particularly present

**Fig. 3:**
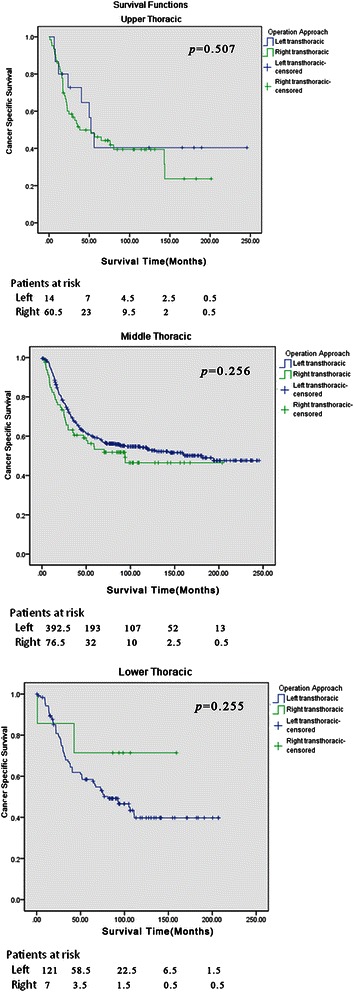
Cancer-specific survival curves stratified by tumor location. When the survival curves were stratified by tumor locations, a significant difference was not revealed between the two groups

3- and 5-year CSS rates were 62.0 % and 44.0 % in the left group, while the corresponding figures in the right group were 56.0 % and 40.0 % respectively (*P* < 0.05). Similarly, the specific cancer survival for the two groups was significantly different (*P* = 0.045) (Fig. 1). Survival analyses were stratified further in terms of tumor stages, which found that the favorable survival advantage was not particularly present (Fig. 2). When the survival curves were stratified by tumor locations, a significant difference was not revealed between the two groups (Fig. 3). Surgical approaches were regarded as one of the prognostic factors in the univariate analysis (*P* = 0.019). However, this significance could not be confirmed in multivariate Cox regression analysis (*P* = 0.193) (Tables 3 and 4)

**Table 3:** Possible prognostic factors and relative risks

Possible prognostic factors	*P*	Hazard ratio	95 % Confidence interval
Age	0.003	1.108	1.006–1.030
Smoking history	<0.001	1.601	1.273–2.014
Drinking history	<0.001	1.687	1.321–2.155
Duration of surgery	0.039	1.002	1.000–1.003
Blood loss during surgery	0.140	1.001	1.000–1.001
Tumor staging	<0.001	1.470	1.231–1.755
Tumor location	0.249	0.887	0.724–1.087
Operation approach	0.019	1.101	1.016–1.193
Location of anastomosis	0.081	0.808	0.635–1.026
Method of anastomosis	0.731	0.955	0.733–1.244
Complications	0.132	0.812	0.619–1.065

**Table 4:** Survival differences after multivariate Cox proportional hazard regression analysis

Item	*P* by Cox regression	Hazard ratio	95 % Confidence interval
Age	0.011	1.016	1.004–1.028
Smoking history	0.028	1.314	1.030–1.677
Drinking history	0.001	1.556	1.201–2.015
Duration of surgery	0.207	1.001	0.999–1.003
Tumor staging	0.000	1.441	1.205–1.723
Operation approach	0.193	1.064	0.969–1.168

.

## Discussion

Esophageal cancer is one of the leading causes of cancer-related mortality worldwide [1]. The prevalence of esophageal cancer, especially adenocarcinoma, has increased in western countries [2]. In China, the squamous cell carcinoma is the most common histological subtype and accounts for over 95 % of the cases [1].

Radical surgery is still the single most important modality in the multidisciplinary treatment of esophageal cancer. However, there are still many controversies surrounding the surgical resection of esophageal cancer way. No matter which kind of operation method chosen, the most important factoris that the surgical techniques chosen should have the following features: simple operation process, small trauma to the patient, fewer complications after surgery, removal of the tumor or the lymph nodes to the greatest extent. Some surgeons advocated the transhiatal esophagectomy for its lower morbidity and mortality with an inadequate lymphadenectomy. Previous studies showed that the optimum extent of lymph node resection is significantly associated with long-term survival after surgery, the group after transthoracic esophageal resection in cleaning the number of lymph nodes is greater than the transhiatal esophageal resection group [11–13]. Choice of surgical techniques, like three-incision transthoracic esophagectomy with three-field lymph node dissection would depend on the patient’s condition and the surgeon’s individual preference [14]. The surgical approaches include left and right transthoracic approaches brought into the current study are the most common used in China. For tumors located in the middle or lower thoracic esophagus, some surgeons advocated left transthoracic esophagectomy rather than the right one, the left approach has many advantages in the treatment of middle or lower third esophageal carcinoma, especially with regard to the lower incidence of postoperative complications and shorter hospital stay [15, 16]. There were also some researches demonstrate that the Ivor-Lewis procedure can be performed with lower rates of postoperative complications and more lymph node retrieval. Then we want to find the clinical outcomes for lymph node-negative esophageal squamous cell carcinoma (ESCC) [17].

Postoperative mortality for esophageal carcinoma, as reported in the literature, ranges from 0 % to 10 % Septicemia, secondary to anastomotic leak and pneumonia among all the relevant complications, are the primary causes of death. The average morbidity rate was reported to be 40.3 %, varying from 26.1 % to 80.4 %. Overall 5-year CSS rate was revealed to be between 40 % and 50 % [18]. On the other hand, the overall incidence of tumor recurrence is approximately 14 % [19–22]. We only enrolled the ESCC patients without lymph node metastasis in this comparison study, as a more homogeneous population with less confusing parameters would make our conclusion more specific and reliable.

Our study found that postoperative CSS rate was better in the left than the right transthoracic group. When the data were stratified, the survival advantage favoring the left transthoracic approach was only present for stage Ib tumors and tumors in the middle third of the esophagus. These survival differences may have resulted from several factors.

Firstly, related anatomies and surgical exposure are dramatically different between the two approaches. Admittedly, the right transthoracic approach could provide excellent surgical exposure to the esophagus and its drainage lymphatic areas. However, with the left thoracotomy, most of the thoracic field’s lymph nodes could also be accessed; however, there might be some interference from the aortic arch. The manipulation of abdominal field is slightly challenging but still accessible after the diaphragm is widely opened. This technique, if used correctly, could offer satisfactory surgical exposure for sufficient tumor and lymph node resection in a single incision. Some researchers found that lymph node metastasis was more common in the patients with tumors in the middle or lower segments of the esophagus, and the metastases were disseminated all the way from the cervical to abdominal areas [23]. Therefore, adequate exposure and resection are critical for ESCC patients.

Secondly, surgical time was significantly different between the left and right transthoracic approaches, with the mean time of 189 and 271 min, respectively (*P* < 0.001). It could be easily understood that the need for re-positioning the patients and two or three incisions would naturally make the right transthoracic surgery longer in the operation time. The drawback of prolonged surgical duration, if any, would mean the exposure of patients to higher anesthesia-related risks.

Moreover, the current study showed that postoperative complication rates were higher in the right group than those in the left group (26.7 % vs. 13.4 %, *P* < 0.001). We found that the incidence of anastomotic leak, incision infection, and respiratory complications were more common in the right transthoracic group. More incisions in the right transthoracic approach would result in more severe post-operative pain and analgesia that is more intensive, which in turn would increase the chances of infection. Anastomosis is more frequently constructed in the upper thorax or the neck in the right transthoracic approach, which may contribute to a higher tension on the anastomosis. However, the occurrence of complications is multi-factorial, like acute inflammatory reaction and anesthetic factors; therefore, further research needs to be carried out before we can substantiate our claim.

According to the outcomes of univariate and multivariate Cox regression analyses, the surgical approach is one of the factors that significantly influences prognosis.

The strengths and limitations of our study should be considered while interpreting these results. The strengths include a large sample of consecutive patients from a well-maintained database and an efficient record system containing abundant tumor information such as tumor grade and stage. Furthermore, we conducted detailed stratified analyses regarding tumor stages and locations in our study, and multivariate analysis was used to explore potential impact factors.

A retrospective study, even when well designed, would inevitably have limitations. Our study included only patients with ESCC and excluded those with esophageal adenocarcinoma. This indicates that our conclusion would not be applicable in the regions where the esophageal adenocarcinoma is prevalent. Because all the surgeries were completed before the publication of AJCC staging manual (7th Edition), it was challenging to verify the exact tumor locations according to the new criteria suggested by this version of staging manual. We could only roughly estimate the tumor locations by categorizing the distances from the superior incisor to esophageal lesions recorded in gastroscopy, without given consideration to some factors like patient’s height.

## Conclusions

In conclusion, after a careful comparison, we found that the left transthoracic approach is not in anyway inferior to the right transthoracic. Our study revealed that it is superior, in some aspects, to the right transthoracic approach regarding surgical and oncological outcomes in the treatment of lymph node negative ESCC.
